# This Is Your Journal

**Published:** 1916-04

**Authors:** 


### This Is Your Journal.

There is but one purpose toward which this journal constantly works, and that is to make it distinctively the journal of the medical men and women who read it,—your journal. Perhaps to be entirely frank it might be well to state that, in order that it may do the greatest amount of good for the greatest number, it is also desired to have it reach as many physicians as possible. If it can increase its subscription list fivefold it can do at least five times the good it now accomplishes.

The editor is encouraged to believe that the physicians who read the TEXAS MEDICAL JOURNAL like it—they frequently write and say so. Those who once get on the subscription list nearly always stay there; this has been especially true in the last few years. Letter after letter comes to the office stating that the JOURNAL is indispensable. Such letters make the editor feel good, for life would be very empty if one could not feel that his or her efforts were resulting in good.

The praise is not due altogether to the editor, either, for she only assembles what others say, except for the few editorials she writes, but it is largely due to the physicians and surgeons who respond so generously to the editor's request for good copy. If you will occasionally, while writing to the editor, also drop a note to the contributor whose article proves helpful to you, that will enable the JOURNAL to get more helpful articles from that contributor. Even a busy doctor likes commendation for what he tries to do for other members of the profession.

Then, if you like the TEXAS MEDICAL JOURNAL, please say so not only to the editor, but to other physicians and surgeons who may or may not be subscribers. If it helps you it would also help them; if you like it, they might also like it.

You might even mention it in your county and district meetings, and thus help to keep it before the physicians. If it could only reach the entire membership of the medical profession in Texas and the Southwest its usefulness would be greatly increased.

Another request: if you think of any way in which your JOURNAL can be made better, will you not sit down and write the editor about it? Your suggestion may not be adopted, but it will be appreciated. Let's work together for our common good. Will you help whenever you can do so without great trouble or inconvenience to yourself?

---

Dr. and Mrs. I. E. Colgin of Waco are at home from Chicago.

